# 3D-Printable and Enzymatically Active Composite Materials Based on Hydrogel-Filled High Internal Phase Emulsions

**DOI:** 10.3389/fbioe.2020.00713

**Published:** 2020-07-21

**Authors:** Lukas Wenger, Carsten P. Radtke, Jacqueline Göpper, Michael Wörner, Jürgen Hubbuch

**Affiliations:** ^1^Institute of Functional Interfaces, Department of Bioengineering and Biosystems, Karlsruhe Institute of Technology, Eggenstein-Leopoldshafen, Germany; ^2^Institute of Engineering in Life Sciences, Section IV: Biomolecular Separation Engineering, Karlsruhe Institute of Technology, Karlsruhe, Germany

**Keywords:** 3D printing, bioprinting, cure-on-dispense, hydrogels, enzymes, beta-galactosidase, biocatalytic reactors, high internal phase emulsions

## Abstract

The immobilization of enzymes in biocatalytic flow reactors is a common strategy to increase enzyme reusability and improve biocatalytic performance. Extrusion-based 3D bioprinting has recently emerged as a versatile tool for the fabrication of perfusable hydrogel grids containing entrapped enzymes for the use in such reactors. This study demonstrates the suitability of water-in-oil high internal phase emulsions (HIPEs) as 3D-printable bioinks for the fabrication of composite materials with a porous polymeric scaffold (polyHIPE) filled with enzyme-laden hydrogel. The prepared HIPEs exhibited excellent printability and are shown to be suitable for the printing of complex three-dimensional structures without the need for sacrificial support material. An automated activity assay method for the systematic screening of different material compositions in small-scale batch experiments is presented. The monomer mass fraction in the aqueous phase and the thickness of printed objects were found to be the most important parameters determining the apparent activity of the immobilized enzyme. Mass transfer limitations and enzyme inactivation were identified as probable factors reducing the apparent activity. The presented HIPE-based bioinks enable the fabrication of flow-optimized and more efficient biocatalytic reactors while the automated activity assay method allows the rapid screening of materials to optimize the biocatalytic efficiency further without time-consuming flow-through experiments involving whole printed reactors.

## 1. Introduction

Biocatalysis is the key to a variety of biotechnological applications, ranging from large-scale industrial processes like the production of high fructose corn syrup (Kirk et al., 2002) to more sophisticated analytical methods like biosensors (Hasan et al., 2014; Rocchitta et al., 2016). Enzymes act as catalysts even at mild reaction conditions. Due to their high chemo- and regioselectivity, they are suitable for the production of high-value products like enantiomerically pure chiral compounds (Nestl et al., 2014). Their usually high cost makes it desirable to immobilize enzymes in order to enhance their stability and shelf life and improve reusability by preventing enzyme-loss (Krishnamoorthi et al., 2015). Immobilized enzymes can be efficiently employed in continuous processes, e.g., using perfusable fixed-bed reactors (Mohapatra and Hsu, 2000; Rodriguez-Colinas et al., 2016; Zhao et al., 2019). While a large variety of immobilization methods like covalent bonding to particles or monolithic support materials does exist, the entrapment of enzymes in hydrogels offers a very straight-forward, effective, and universally applicable route (Krishnamoorthi et al., 2015; Schmieg et al., 2019). Hydrogels are hydrophilic polymer networks with a high water content that are well-suited to accommodate cells and proteins in an aqueous environment (Hoffman, 2012). The mesh size of the hydrogel polymer network can be tuned (Hagel et al., 2013; Rehmann et al., 2017) in order to ensure the retention of the usually relatively large enzymes while still allowing the diffusion of smaller substrate and product molecules through the material (Krishnamoorthi et al., 2015). Besides increased cost efficiency, the immobilization of enzymes offers a way to realize processes combining different spatially separated reactions. These compartmentalized enzymatic cascades may prevent undesirable effects like product inhibition or cross-reactivities (Rabe et al., 2017). A drawback of entrapping enzymes in hydrogels is the reduced mass transfer through the hydrogel matrix which lowers the apparent biocatalytic activity as compared to the freely dissolved enzyme (Krishnamoorthi et al., 2015; Schmieg et al., 2018). This limitation can be counteracted by increasing the surface-area-to-volume ratio of the hydrogel structures (Schmieg et al., 2018).

Extrusion-based bioprinting methods can be used as a simple tool to produce hydrogel structures in customized 3D shapes, typically laden with living cells or bioactive molecules (Groll et al., 2016; Ozbolat and Hospodiuk, 2016). The so-called bioink, a hydrogel precursor formulation blended with the biological component, is dispensed layer-by-layer from a cartridge through a nozzle and usually cross-linked after extrusion in order to retain a stable 3D shape (Hölzl et al., 2016; Panwar and Tan, 2016). Extrusion-based printing is widely employed in biofabrication but resolutions are usually low with extruded strand widths of typically 200 μm to 1,000 μm (Hölzl et al., 2016). Achievable strand widths and hence printing quality and resolution depend largely on the rheological properties of the bioink. Shear-thinning behavior is desirable to facilitate the material flow through the nozzle and allow dispensing at lower pressure (Melchels et al., 2014). After extrusion, the ink should preserve its shape without spreading until fixation is achieved by cross-linking. This is ensured by a sufficiently high viscosity or the presence of a yield stress (Malda et al., 2013; Hölzl et al., 2016; Mouser et al., 2016). However, many hydrogel precursor solutions are low-viscosity liquids that lack the necessary rheological properties. Strategies to enhance printability include improving the rheological properties of the ink by the addition of viscosity-enhancing additives (Markstedt et al., 2015; Schmieg et al., 2018) or by pre-crosslinking (Skardal et al., 2010; Rutz et al., 2015). Other approaches aim at improving shape preservation after extrusion by printing into a support bath (Wu et al., 2011; Hinton et al., 2015) or minimizing the time between extrusion and final fixation of the shape in order to reduce spreading of the material. Depending on the crosslinking mechanism, this can be achieved by co-extruding a crosslinking agent through a coaxial nozzle (Colosi et al., 2016; Jia et al., 2016) or by *in situ* photopolymerization using a cure-on-dispense setup (Hockaday et al., 2012; Sears et al., 2016; Maeng et al., 2019).

High internal phase emulsions (HIPEs) are paste-like emulsions containing more than 74% (v/v) of internal phase (Cameron et al., 2011). Below the yield stress, they act as a solid retaining their shape. Applying a force above the yield stress initiates the flow of the material (Foudazi et al., 2015). This property makes HIPEs ideal candidates for extrusion-based 3D printing (Sears et al., 2016, 2019). The external phase of HIPEs can be polymerized creating so-called polyHIPEs, a monolithic and porous polymer scaffold with void sizes typically in the range of one to several hundred micrometers (Hainey et al., 1991; Sergienko et al., 2002; Silverstein, 2017) and interconnecting pores between the voids (Silverstein, 2017). There are two approaches toward formulating low-viscosity hydrogel precursor solutions as printable HIPE-based inks: Using oil-in-water HIPEs with a polymerizable external phase (Sears et al., 2019) or using water-in-oil HIPEs with both phases being polymerizable (Gitli and Silverstein, 2008, 2011; Kovačič et al., 2011). The first approach yields a hydrogel filled with droplets of liquid oil, while the second approach yields a typical open-cell polyHIPE scaffold filled with hydrogel.

In (bio-)chemical engineering, 3D printing methods can enable the fabrication of highly sophisticated, geometrically optimized reactors not producible by conventional methods (Parra-Cabrera et al., 2018), as has been shown for bed geometries of chromatography columns (Fee et al., 2014), heat exchangers (Fee, 2017) or microfluidic reactors (Konarova et al., 2017). 3D printing can also dramatically speed up the production of prototypes (Ngo et al., 2018), allowing the iterative testing of different designs. Kazenwadel et al. (2016) used a commercial inkjet 3D printer to manufacture biocatalytic flow reactors based on a 3D-printed grid of synthetic material. Glucose oxidase and horseradish peroxidase were covalently immobilized on the surface of the grid in a subsequent step. This concept has been developed further by directly printing enzyme-containing hydrogel grids in a single step using extrusion-based 3D bioprinters (Maier et al., 2018; Schmieg et al., 2018; Peng et al., 2019). Enzymatically active printed hydrogels with living yeast cells have been reported as well (Saha et al., 2018).

**Figure 1:**
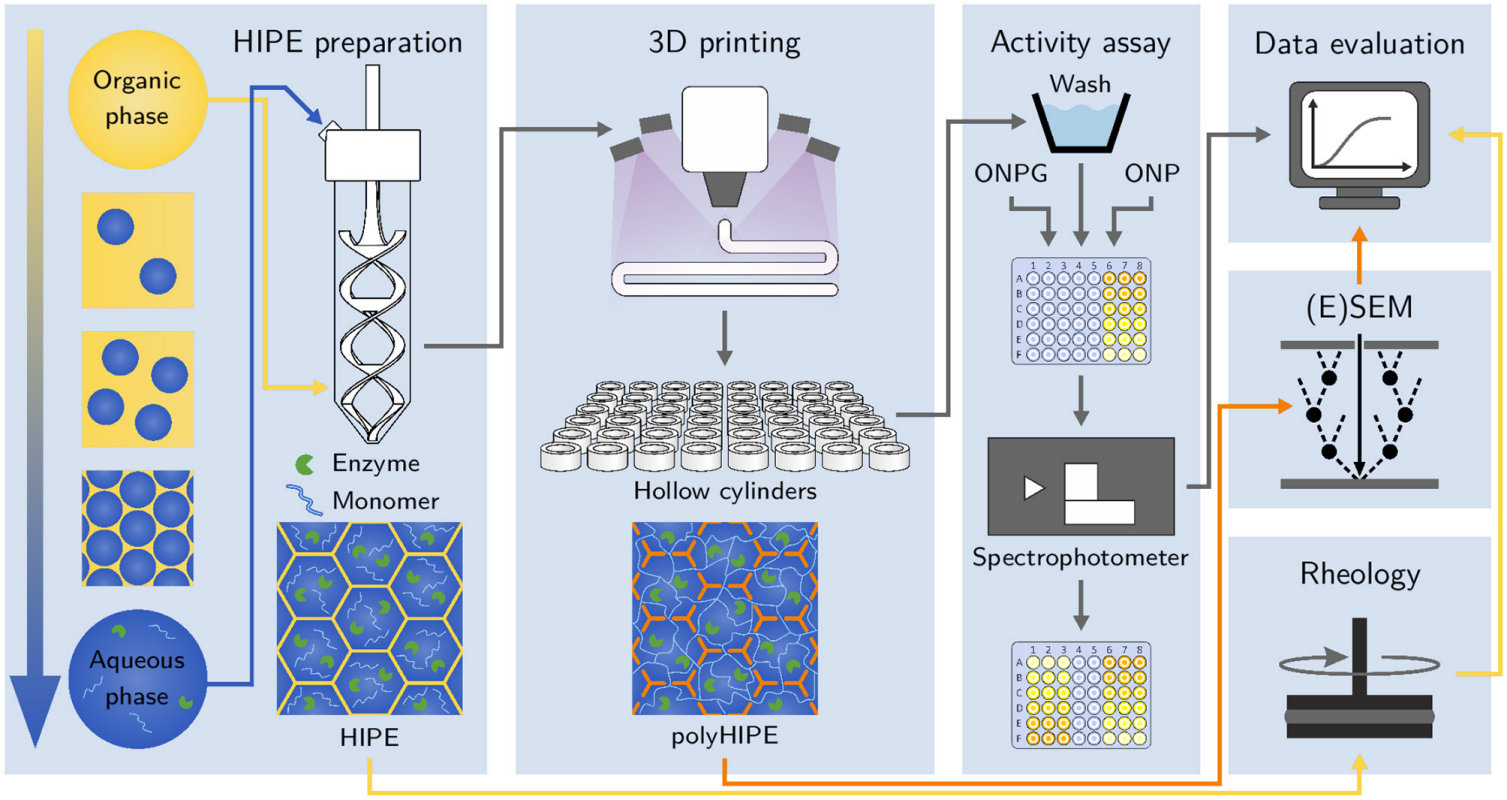
Schematic of the workflow applied in this study. After preparation, HIPEs are printed and photopolymerized *in situ* using a cure-on-dispense setup. The resulting hydrogel-filled polyHIPE cylinders are analyzed in automated activity assays in a 48-well microtiter plate format. Samples of liquid HIPEs are analyzed using rheological measurements. (Environmental) scanning electron micrographs of polyHIPEs are taken to analyze their morphology and confirm the presence of hydrogel inside the polyHIPE voids.

The reported methods and inks for extrusion-based 3D printing of enzyme-containing hydrogels are limited to the fabrication of very basic geometries comprising only a few layers and exhibiting relatively high wall thicknesses, thereby limiting mass transfer and reducing efficiency. Furthermore, a systematic investigation of the influence of material parameters on the resulting apparent activity of the printed hydrogels is missing. The study presented here investigates the approach to enhance the printability of low-viscosity hydrogel precursor solutions using water-in-oil HIPEs as bioinks with excellent printability enabling the fabrication of more complex structures. Scanning electron microscopy techniques are used to analyze the scaffold morphology and confirm the formation of hydrogel inside the polyHIPE voids. The enzyme β-galactosidase is entrapped in the hydrogel-filled polyHIPEs and an automated activity assay method is presented to determine the influence of different HIPE compositions on the apparent activity of the produced materials. A workflow of the presented study is shown in Figure 1.

## 2. Materials and Methods

### 2.1. Chemicals

All chemicals were purchased from Sigma Aldrich and used as received. For the organic phase of the HIPEs, 2-ethylhexyl acrylate (EHA), isobornyl acrylate (IBOA), trimethylolpropane triacrylate (TMPTA), poly(ethylene glycol)-block-poly(propylene glycol)-block-poly(ethylene glycol) (Pluronic® L-121), and diphenyl(2,4,6-trimethylbenzoyl)phosphine oxide (Darocur® TPO) were used. The aqueous phase contained acrylic acid (AA), poly(ethylene glycol) diacrylate with an average molar mass of *M*_*n*_= 700 g/mol (PEG-DA 700), lithium phenyl-2,4,6-trimethylbenzoylphosphinate (LAP) and β-galactosidase from *Aspergillus oryzae* (EC 3.2.1.23, 10.9 units/mg). Activity assays were conducted using 2-nitrophenyl β-D-galactopyranoside (ONPG) as the substrate and calibration curves of the reaction product were determined from serial dilutions of 2-nitrophenol (ONP). All buffers and solutions were prepared with ultrapure water from a Purelab Ultra water purification system (Elga, High Wycombe, UK) and filtered through an 0.2 μm cellulose acetate filter (Sartorius AG).

### 2.2. Stock Solutions

20% (w/w) AA and 20% (w/w) PEG-DA 700 were prepared as stock solutions for the aqueous phase, both buffered with 100 mM phosphate and adjusted to pH 7. A stock solution of 1.6 kU/mL β-galactosidase was prepared in 100 mM phosphate (pH 7). Serial dilutions of ONPG for the activity assays and ONP for the calibration curves were prepared in 100 mM phosphate, pH 7. The stock solutions of β-galactosidase, ONPG and ONP were frozen in aliquots at -30 °C and thawed directly before use.

### 2.3. Preparation of HIPEs

A series of preliminary experiments resulted in the selection of a set of suitable chemicals for HIPE preparation. The external organic phase of the HIPEs was prepared from the monomers EHA, IBOA, and TMPTA, the surfactant Pluronic® L-121 and the photoinitiator Darocur® TPO. For all HIPEs, the mass ratio between EHA, IBOA, and TMPTA was kept constant at 2.3:4.2:1 while the amount of Pluronic® L-121 in the organic phase was varied between 6 and 12% (w/w). The Darocur® TPO concentration was 0.5% (w/w) for all HIPEs. All compositions of organic phase are listed in Table S1. The internal aqueous phase was always freshly prepared from buffered stock solutions of AA, PEG-DA 700, LAP and β-galactosidase. The resulting aqueous phase had constant concentrations of LAP (1 mg/mL) and β-galactosidase (40 units/mL) with varying total monomer mass fractions and a constant molar ratio of AA:PEG-DA 700 = 10:1. In HIPEs with a lower monomer content in the aqueous phase than 14% (w/w), a corresponding amount of AA stock solution was replaced by a buffered solution of 100 mM phosphate at pH 7 that was adjusted to the same conductivity as the AA stock solution by adding NaCl. The corresponding amount of PEG-DA 700 solution was replaced by 100 mM phosphate buffer, pH 7. All HIPE variations prepared in this study, as listed in Table 1, are derived from HIPE A, a HIPE with 14% (w/w) monomer in the aqueous phase, 12% (w/w) surfactant in the organic phase, 87.5% aqueous phase volume fraction and printed using a 250 μm nozzle.

**Table 1:** Overview of the HIPEs prepared and printed in this study.

	**Varied parameter**	**Monomer in aqueous phase [% (w/w)]**	**Surfactant in organic phase [% (w/w)]**	**Aqueous phase volume fraction [% (v/v)]**	**Nozzle diameter (μm)**
HIPE A	—	14	12	87.5	250
HIPE variations	Monomer	10.5	12	87.5	250
		7	12	87.5	250
		3.5	12	87.5	250
		1.75	12	87.5	250
		0	12	87.5	250
	Surfactant	14	9	87.5	250
		14	6	87.5	250
	Aqueous phase	14	12	90	250
		14	12	85	250
		14	12	82.5	250
		14	12	80	250
	Nozzle diameter	14	12	87.5	840
		14	12	87.5	110

*The table shows the mass fraction of monomer in the internal aqueous phase, the surfactant mass fraction in the external organic phase, the aqueous phase volume fraction and the nozzle diameter used for printing hollow cylinders for activity assays. All variations are derived from HIPE A (blue). Parameters deviating from HIPE A are highlighted in orange*.

**Figure 2:**
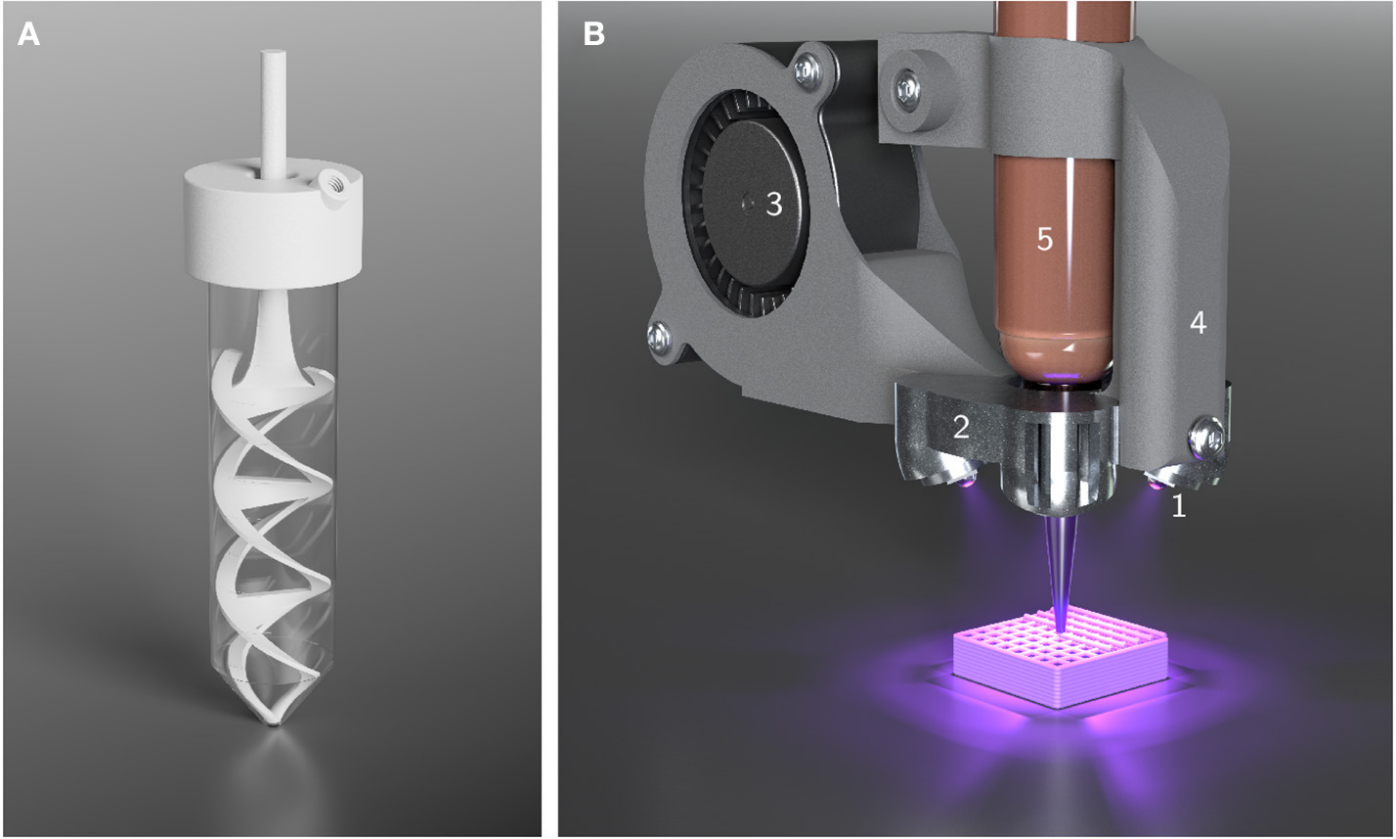
Illustrations of custom-made equipment used for HIPE preparation and printing. **(A)** Setup used for HIPE emulsification. The helical stirrer blade and the lid were custom-made for 50 mL Falcon tubes. The lid offers an opening for the stirrer blade and a threaded hole to connect tubing and allow the continuous addition of aqueous phase using a syringe pump. **(B)** Cure-on-dispense setup used in combination with a Gesim BioScaffolder 3.1. Four high power UV LEDs (1) are attached to a metal heatsink (2) which is perfused by air from a fan (3) for active cooling. The mount (4) allows the direct attachment to 10 mL cartridges (5).

Emulsification of the HIPEs was performed in 50 mL Falcon tubes (Corning, Inc.) using an overhead stirrer (Velp Scientifica DLS) and a specifically designed, 3D-printed, helical stirrer blade made from nylon, as depicted in Figure 2A. For HIPEs with a percentage of 87.5% (v/v) of aqueous phase, 2.5 mL of organic phase were added to a Falcon tube. Under continuous stirring, 17.5 mL of aqueous phase were continuously added at a rate of 1.25 mL/min using a syringe pump (Cetoni neMESYS 290N). The stirring rate was initially set to 600 rpm and increased to 800 rpm after 2 min and 1000 rpm after 7 min. After addition of the aqueous phase, agitation was maintained for another 5 min at 1000 rpm. For HIPEs with a percentage of aqueous phase different from 87.5%, the amounts of organic phase and aqueous phase were adjusted to always produce 20 mL of HIPE in total. The addition rate and time points of stirring rate changes were adjusted relative to the amount of organic phase, as shown in Table S2.

### 2.4. Rheology

Rheological measurements of HIPEs were performed using an MCR 301 rheometer (Anton Paar GmbH). A measurement geometry with profiled parallel plates with a diameter of 25 mm and a gap width of 150 μm was used to perform shear stress-controlled rotational tests. Yield stress values were determined by plotting deformation over shear stress in a log-log graph, fitting the two linear regions of the plot with tangents and calculating their point of intersection.

Using the same experimental setup, oscillatory measurements with controlled shear stress in a range of 1 Pa to 1000 Pa were performed at an angular frequency of ω = 10/s. The storage modulus *G*′ and loss modulus *G*″ were recorded. All measurements were carried out as technical triplicates.

### 2.5. (Environmental) Scanning Electron Microscopy (ESEM and SEM)

For sample preparation, HIPEs were polymerized on microscope slides in a frame of 2 mm in height. The polymerization was carried out for 1 min from each side at an intensity of approximately 25 mW/cm^2^ and a peak wavelength of 365 nm.

Samples for environmental scanning electron microscopy (ESEM) were stored in 100 mM phosphate buffer (pH 7) and cut immediately before analysis to investigate the cross-section. ESEM micrographs were taken using an FEI Quanta 650 FEG (Thermo Fisher Scientific, Inc.) in a water-saturated atmosphere at a pressure between 704 and 823 Pa, a working distance between 6.8 mm and 8.1 mm, a 2,000-fold magnification and an acceleration voltage of 15 kV.

For scanning electron microscopy (SEM), samples were freeze-dried, cut and the cross-section coated with platinum. Analysis was carried out on a Tescan Vega 3 SBU at an acceleration voltage of 8 kV and a working distance of 14 mm.

### 2.6. 3D Printing

HIPEs were 3D-printed employing two different extrusion-based printers: a piston-driven, previously described modified Ultimaker Original+, introduced as *Biomaker* by Radtke et al. (2017), and a pneumatically driven Gesim BioScaffolder 3.1. Both printers were equipped with a cure-on-dispense setup similar to a system reported by Sears et al. (2016), based on four high-power UV LEDs emitting at a peak wavelength of 365 nm (LEDengin, San Jose, USA). The setup employed in combination with the BioScaffolder is depicted in Figure 2B.

For activity assays, simple hollow cylinders with a diameter of 8 mm and a height of 2.4 mm were printed, designed to fit into the wells of 48-well microtiter plates (**Figure 5C**). The *Biomaker* was used in combination with conical nozzles with an inner diameter of 250 and 840 μm to print 300 μm layers while curing at an intensity of approximately 3.5 mW cm^-2^. The BioScaffolder was equipped with Micron-S nozzles (Vieweg GmbH) with an inner diameter of 110 and 100 μm layers were printed while curing at an intensity of approximately 13 mW cm^-2^. In order to prevent clogging, aluminum foil was wrapped around the end of the nozzle to shield the nozzle opening from UV irradiation. All printing parameters are summarized in Table 2. After the printing process, all cylinders were post-cured at an intensity of approximately 25 mW cm^-2^ for 2 min.

**Table 2:** Printing parameters employed in the fabrication of polyHIPE cylinders and exemplary prints.

**Printer**	**Printing diameter speed (mm/s)**	**Nozzle type (μm)**	**Nozzle height –**	**Layer pressure (μm)**	**Extrusion rate (kPa)**	**Extrusion (EU^a^/mm)**	**UV intensity (mW cm^−2^)**
Biomaker	9	840	conical	300	–	0.0009	3.5
Biomaker	9	250	conical	300	–	0.0006	3.5
Bioscaffolder	9	110	Micron-S	100	30 ± 10	–	13.0

a *EU, extrusion unit, an arbitrary unit to adjust the extrusion rate on the Biomaker*.

### 2.7. Activity Assays With Printed PolyHIPEs

**Figure 3:**
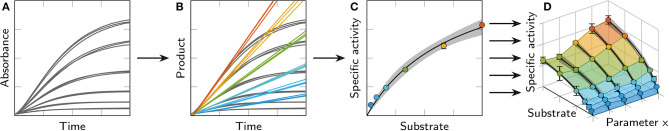
Overview of the data processing steps used for the evaluation of activity assays with polyHIPE cylinders. The example shows data from the analysis of different aqueous phase volume fractions. **(A)** The product formation was monitored by measuring the absorbance at 460 nm in a spectrophotometer. The curves show triplicates of six different substrate concentrations. **(B)** Using calibration curves, the product concentration was calculated from the absorbance signal and the maximum slope of the resulting curves was calculated to determine the maximum volumetric activity. Due to diffusion limitations, the maximum slope does not occur at the start of the reaction but after an initial delay time *t*_*delay*_. **(C)** The specific activity was calculated from the volumetric activity and the resulting data points were plotted over the substrate concentration and fitted with a Michaelis-Menten equation (95% confidence intervals shown in gray). **(D)** Several kinetics curves were combined with a surface plot to compare different conditions.

PolyHIPE cylinders, printed as described above, were washed three times for 5 min: once in 30% (v/v) ethanol in 100 mM phosphate (pH 7) and twice in 100 mM phosphate (pH 7). The washed cylinders were transferred into a 48-well microtiter plate, one cylinder per well. ONPG solutions at concentrations between 1 mM and 30 mM in 100 mM phosphate (pH 7) were added using a Tecan Freedom Evo pipetting platform and the conversion from ONPG to ONP catalyzed by β-galactosidase was monitored online at a wavelength of 460 nm for 90 min using a Tecan infinite M200 pro spectrophotometer (Figure 3A). All activity assays were carried out at 25 °C. Employing calibration curves, ONP concentrations were calculated from the absorbance signal and the maximum volumetric activity per well was calculated by determining the maximum slope of each curve which occurred after a variable delay time *t*_*delay*_ (Figure 3B). The specific activity of each sample was determined by dividing the volumetric activity by the amount of enzyme in one polyHIPE cylinder. Plots of the specific activity over the substrate concentration were fitted with Michaelis-Menten kinetics (Figure 3C). Combining several of these kinetics resulted in 3D plots showing the influence of both substrate concentration and a second varied parameter on the specific activity (Figure 3D).

### 2.8. Calibration Curves and Equilibration Time

Data for ONP calibration curves for the polyHIPE activity assays were collected following the same procedure as applied for the activity assays, only adding solutions of up to 25 mM ONP instead of ONPG before monitoring the absorbance at 460 nm for 90 min. Due to diffusion and evaporation effects, the observed absorbance was not constant over time but showed an exponential decay at the beginning, turning into a linear decay after a certain time. The recorded data for the calibration curves of HIPE A are shown as an example in the (Figure S1).

Linear calibration curves were determined from the end points of the measurements where an equilibrium between polyHIPE cylinder and supernatant was already reached. Triplicates of five different ONP concentrations (2.5 mM to 20 mM) were used as the calibration points. To determine the equilibration time as a measure of diffusion limitations, an algorithm was employed that calculated the earliest point of time at which the slopes of the measured absorbance curves were within 20% of the end slope of the curves. The time span from the addition of ONP until this time point was defined as the equilibration time (see Figure S1).

### 2.9. Enzyme Leaching

To determine the amount of leached enzyme during different process steps, activity assays were performed with the supernatants of the polyHIPE cylinder wash procedure described above. Also, single polyHIPE cylinders were incubated with 300 μl phosphate buffer (100 mM, pH 7) for 90 min mimicking the polyHIPE activity assay to analyze the amount of leached enzyme during the whole incubation period. The supernatant activity assays were carried out in 96-well microtiter plates by adding 150 μl 30 mM ONPG in 100 mM phosphate (pH 7) to a 50 μl sample of supernatant (dilution factor *f* = 4) and recording the conversion of ONPG to ONP at a wavelength of 460 nm for 20 min. In order to account for samples from different wash solutions (30% ethanol and 100 mM phosphate), separate calibration curves with a range of ONP and β-galactosidase concentrations were recorded for the respective samples and used to calculate the volumetric activity of the samples and the amount of active enzyme leached from the polyHIPE cylinders. For error analysis of the polyHIPE activity assays (see following section), the volumetric activity of the supernatant samples after a 90 min incubation period (*v*_*leached*, 90 min_) was determined taking into account the dilution factor *f*.

### 2.10. Error Analysis of PolyHIPE Activity Assays

An error analysis was performed in order to estimate the influence of leached enzyme on the activity assays performed with printed polyHIPEs. The maximum error *E*_*max*_ and minimum error *E*_*min*_ were calculated by introducing a best-case and a worst-case scenario. These scenarios assume that the total apparent volumetric activity *v*_*apparent*_ observed in the polyHIPE activity assays is composed of a proportion caused by the enzyme immobilized in the polyHIPEs (*v*_*immobilized*_) and a proportion caused by leached enzyme (*v*_*leached*_). The error *E* is defined as

(1) $$\begin{array}{l} E=\frac{v_{leached}}{v_{apparent}}. \end{array}$$

The worst-case scenario assumes that *v*_*leached, max*_ equals the volumetric activity in the supernatant after a 90 min incubation period (*v*_*leached*, 90 min_).

(2) $$\begin{array}{l} v_{leached,max}=v_{leached,\text{90}\;\text{min}} \end{array}$$

The best-case scenario assumes that enzyme is leaching from the polyHIPE at a constant rate over 90 min and accumulates in the supernatant of the polyHIPE cylinders. As the maximum volumetric activity of the polyHIPE cylinders occurs after an initial delay time *t*_*delay*_ (see Figure 3, the amount of leached enzyme that has accumulated during this time period determines *v*_*leached, min*_.

(3) $$\begin{array}{l} v_{leached,min}=\frac{t_{delay}}{\text{90}\;\text{min}}\cdot v_{leached,\text{90}\;\text{min}} \end{array}$$

These scenarios result in the following definitions of maximum and minimum error.

(4) $$\begin{array}{l} E_{max}=\frac{v_{leached,\text{90}\;\text{min}}}{v_{apparent}} \end{array}$$

(5) $$\begin{array}{l} E_{min}=\frac{t_{delay}\cdot v_{leached,\text{90}\;\text{min}}}{\text{90}\;\text{min}\cdot v_{apparent}} \end{array}$$

### 2.11. Statistical Analysis

The statistical significance of data was tested employing a one-way analysis of variance (ANOVA) and the Tukey method for multiple comparisons.

## 3. Results

### 3.1. Rheology

**Figure 4:**
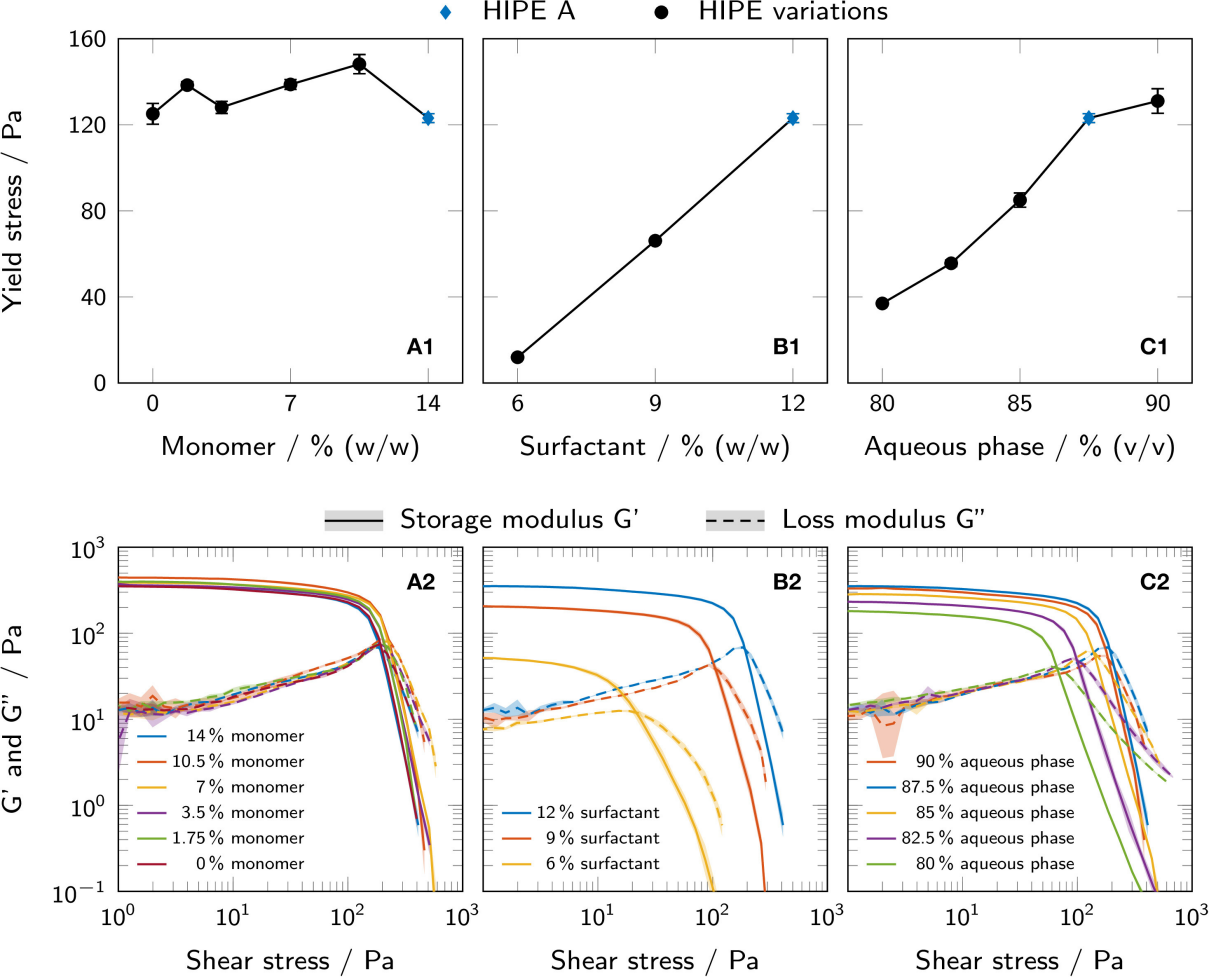
**(A1–C1)** Yield stress of all prepared HIPEs, as determined by rotational tests and the tangent fit method. The results are presented as mean values ± standard deviation (*n* = 3, technical triplicates). **(A2–C2)** Shear stress-controlled oscillatory measurements showing storage and loss moduli of all prepared HIPEs. The results are presented as mean values and standard deviations are indicated by shaded areas (*n* = 3, technical triplicates). All variations are derived from HIPE A (depicted in blue in each graph). The influence of **(A)** monomer mass fraction in the aqueous phase, **(B)** surfactant mass fraction in the organic phase and **(C)** aqueous phase volume fraction is shown.

Rheological measurements were performed in order to investigate the influence of changing HIPE compositions on yield stress and storage and loss moduli as important parameters for printability. Figure 4 shows both yield stress and storage and loss moduli for HIPEs with a varying monomer mass fraction in the aqueous phase (A), surfactant mass fraction in the organic phase (B) and aqueous phase volume fraction (C). Yield stress values could be determined for all prepared HIPEs. The different formulations of aqueous phase not formulated as HIPEs did not exhibit a yield point. Of all parameters tested, the surfactant concentration in the organic phase had the largest impact on yield stress with a 10-fold increase between 6% and 12% (w/w) surfactant. Increasing the aqueous phase volume fraction from 80% to 90% (v/v) led to a 3.5-fold increase in yield stress. For different monomer concentrations in the aqueous phase, the yield stress fluctuated between (123.1 ± 2.1) Pa and (148.2 ± 4.4) Pa without any clear trend.

Similar trends could be observed for the loss and storage moduli in shear stress-controlled oscillatory measurements. All samples showed a higher storage than loss modulus at low shear stress, i. e. a loss factor *tan*(δ) < 1, indicating a gel-like nature of the HIPEs.

### 3.2. Printability

**Figure 5:**
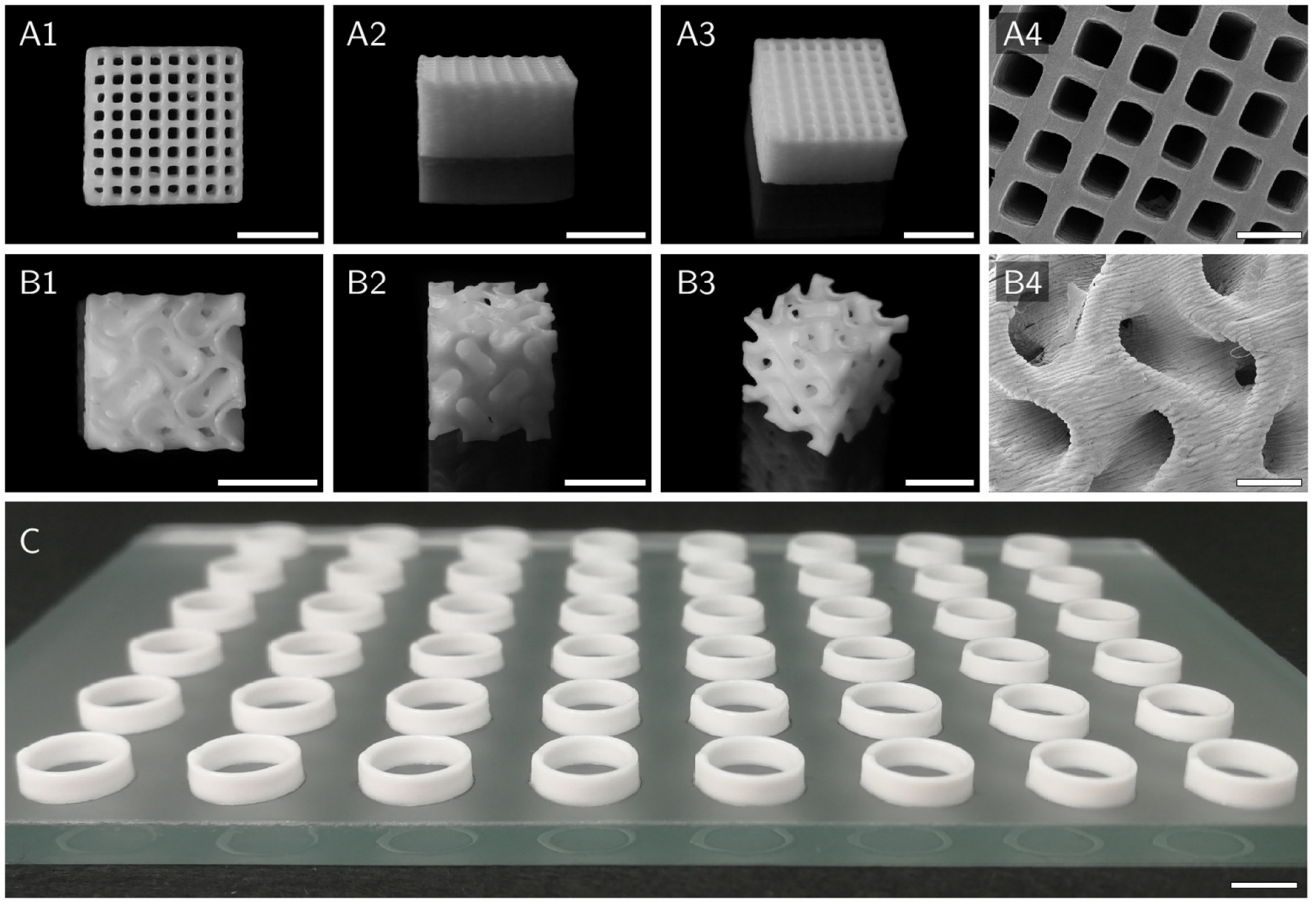
Various scaffolds printed with HIPE A to demonstrate printability. **(A1–A4)** Top, side, angled, and SEM view of a cuboid grid structure with an edge length of 10 mm and a height of 5 mm. **(B1–B4)** Top, side, angled and SEM view of a cubic gyroid structure with an edge length of 8 mm. Model adapted from Vitroid (2018). **(C)** 48 hollow cylinders, as used for activity assays, printed on a glass plate in microtiter plate format. Scale bars represent 5 mm **(A1–A3,B1–B3,C)** and 1 mm **(A4,B4)**, respectively.

Hollow cylinders of different HIPE compositions, as depicted in Figure 5C, were printed for batch activity assays using the *Biomaker*. Samples of printed cylinders made from HIPEs with five different compositions (*n* = 5 × 5 = 25) representing the whole measured yield stress range were weighed to assess how reproducibly cylinders could be printed using identical printing parameters with different HIPEs. ANOVA showed no significant differences between the analyzed groups (*p* = 0.68). The overall mean weight of the printed cylinders was (45.7 ± 0.9) mg. To illustrate the excellent printability of HIPE inks, more complex structures were printed from HIPE A using a pneumatically driven Gesim BioScaffolder 3.1. Figure 5A shows a grid with a 10 mm edge length and a height of 5 mm consisting of 100 μm layers. All inter-strand spaces are free of obstructing artifact making the grid entirely perfusable. The gyroid structure depicted in Figure 5B demonstrates the applicability of HIPEs as inks for prints of more complex reactor geometries including overhangs without the need for sacrificial material. While the printability of all prepared HIPEs was sufficient to print the simple hollow cylinders needed for the activity assays, a certain yield stress was essential for prints of more complex structures as shown in Figure 5.

### 3.3. PolyHIPE Morphology

**Figure 6:**
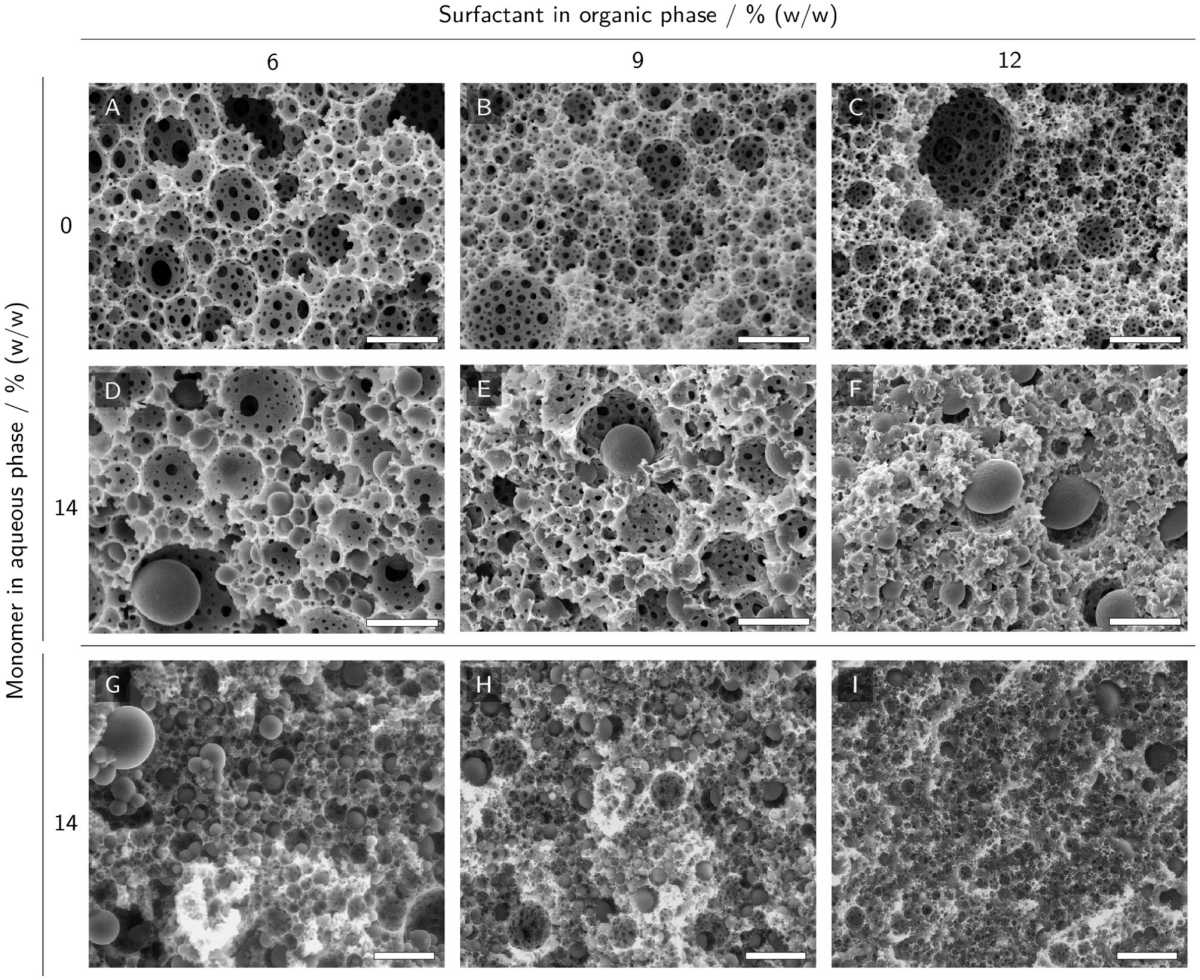
(E)SEM micrographs of polyHIPE cross-sections with 0% and 14% (w/w) monomer in the aqueous phase and 6% to 12% (w/w) surfactant in the organic phase. **(A–C)** SEM micrographs of polyHIPEs without monomer in the aqueous phase display a typical polyHIPE morphology with empty voids and interconnecting pores between the voids. **(D–F)** SEM micrographs of polyHIPEs with monomers in the aqueous phase. **(G–I)** ESEM micrographs of polyHIPEs with monomers in the aqueous phase. The scale bars represent 10 μm **(A–F)** and 25 μm **(G–I)**, respectively.

SEM and ESEM micrographs of polyHIPE cross-sections were taken to investigate the scaffold morphology and the presence of polymerized hydrogel in the voids of the scaffolds. PolyHIPEs with different surfactant mass fractions in the organic phase and no monomers in the aqueous phase (Figures 6A–C) were compared to the corresponding polyHIPEs containing 14% (w/w) monomer in the aqueous phase (Figures 6D–I). HIPEs without monomers in the aqueous phase resulted in samples exhibiting typical polyHIPE morphologies with empty voids and interconnecting pores between the voids (Figures 6A–C). Increasing the surfactant mass fraction in the organic phase resulted in smaller voids. SEM micrographs of polyHIPEs with 14% (w/w) monomer in the aqueous phase are represented in Figures 6D–F. Here, the typical polyHIPE scaffold is only visible in the samples with 6 and 9% (w/w) surfactant in the organic phase. Some of the voids are filled with hydrogel spheres slightly smaller than the surrounding voids. It is noticeable that the interconnecting pores of these samples are smaller and less frequent compared to the samples without monomers in the aqueous phase, resulting in a lower degree of openness of the scaffold. The SEM micrograph of the sample with 12% (w/w) surfactant in the organic phase shows a collapsed scaffold structure (Figure 6F) indicating a reduced mechanical stability of the porous scaffold. As a comparison, the samples containing monomers in the aqueous phase were also analyzed by ESEM (Figure 6G–I) which does not require the samples to be freeze-dried before analysis. Here, not only a few but most voids were filled with hydrogel spheres and even at a surfactant concentration of 12% (w/w) in the organic phase, the ESEM micrographs show an intact polyHIPE scaffold, implying the freeze-drying step to be the cause for the collapsed scaffold observed in SEM. Despite the preparation of the ESEM samples in a hydrated state, the hydrogel spheres were smaller than the surrounding voids implying a certain degree of shrinkage.

In order to analyze the effect of the printing process on the material morphology, additional SEM micrographs were taken of polyHIPEs printed with a 250 μm nozzle and with different surfactant concentrations (see Figure S2). The cross-sections of these samples showed intact polyHIPE scaffolds indicating that the printing process did not impair the structure of the resulting material. Furthermore, micrographs of the surface of these printed samples revealed an open-porous surface potentially suitable for the penetration of the material by substrate solutions. Micrographs of a printed cylinder of HIPE A at a lower magnification showed a homogeneous cross-section without any visible interfaces between different printed layers (see Figure S3).

### 3.4. Equilibration Time

**Figure 7:**
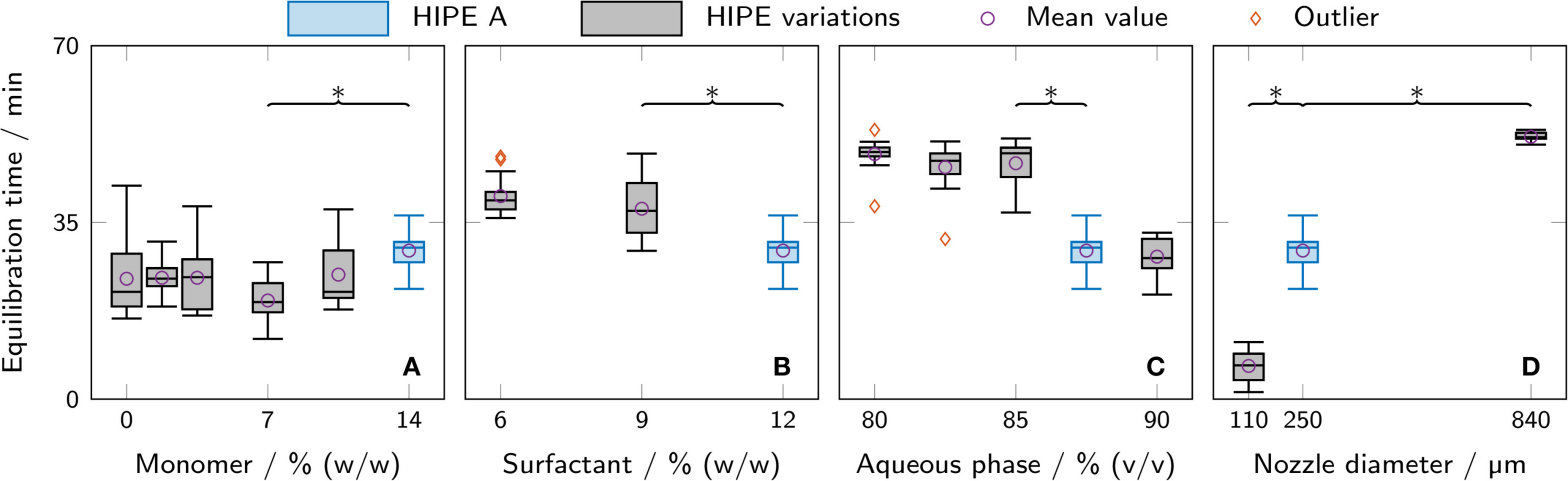
Equilibration time as determined from measurements for ONP calibration curves with different polyHIPE cylinders. All variations are derived from HIPE A printed with a 250 μm nozzle (depicted in blue in each graph). The box plots represent the median and the upper and lower quartile. The whiskers represent the most extreme value still within a 1.5-fold interquartile range (IQR) from the upper and lower quartile, respectively. All data points outside the 1.5-fold IQR are depicted as outliers. Each box represents triplicates of six different ONP concentrations (*n* = 6 × 3 = 18). For clarity, only significant differences to the nearest significantly different data points are highlighted by asterisks (*p* < 0.05). The compared parameters are **(A)** monomer mass fraction in the aqueous phase, **(B)** surfactant mass fraction in the organic phase, **(C)** aqueous phase volume fraction, and **(D)** nozzle diameter.

The equilibration time when adding ONP solutions to polyHIPE cylinders was determined from calibration data as a measure of diffusion limitations. Data for calibration curves were generated by adding serial dilutions of ONP to printed polyHIPE cylinders and recording the absorbance at 460 nm for 90 min. The observed signals showed an exponential decay at the beginning, turning into a linear decay after a certain time span depending on the polyHIPE composition and the thickness of the cylinder. The linear decay of the absorbance signal was caused by evaporation of ONP, as confirmed experimentally. The time span until a steady slope was reached was defined as the equilibration time. It was determined for all measured samples and is shown in Figure 7. The mass fraction of monomer in the aqueous phase was found to have no significant effect within the analyzed range (Figure 7A). Both a higher mass fraction of surfactant in the organic phase and a higher aqueous phase volume fraction correlated with a decrease in equilibration time (Figures 7B,C). The highest impact of all tested parameters was observed for the nozzle diameter where the equilibration time increased from (6.6 ± 3.1) min to (52.0 ± 0.9) min when changing nozzle diameters from 110 μm to 840 μm.

### 3.5. Enzyme Leaching

**Figure 8:**
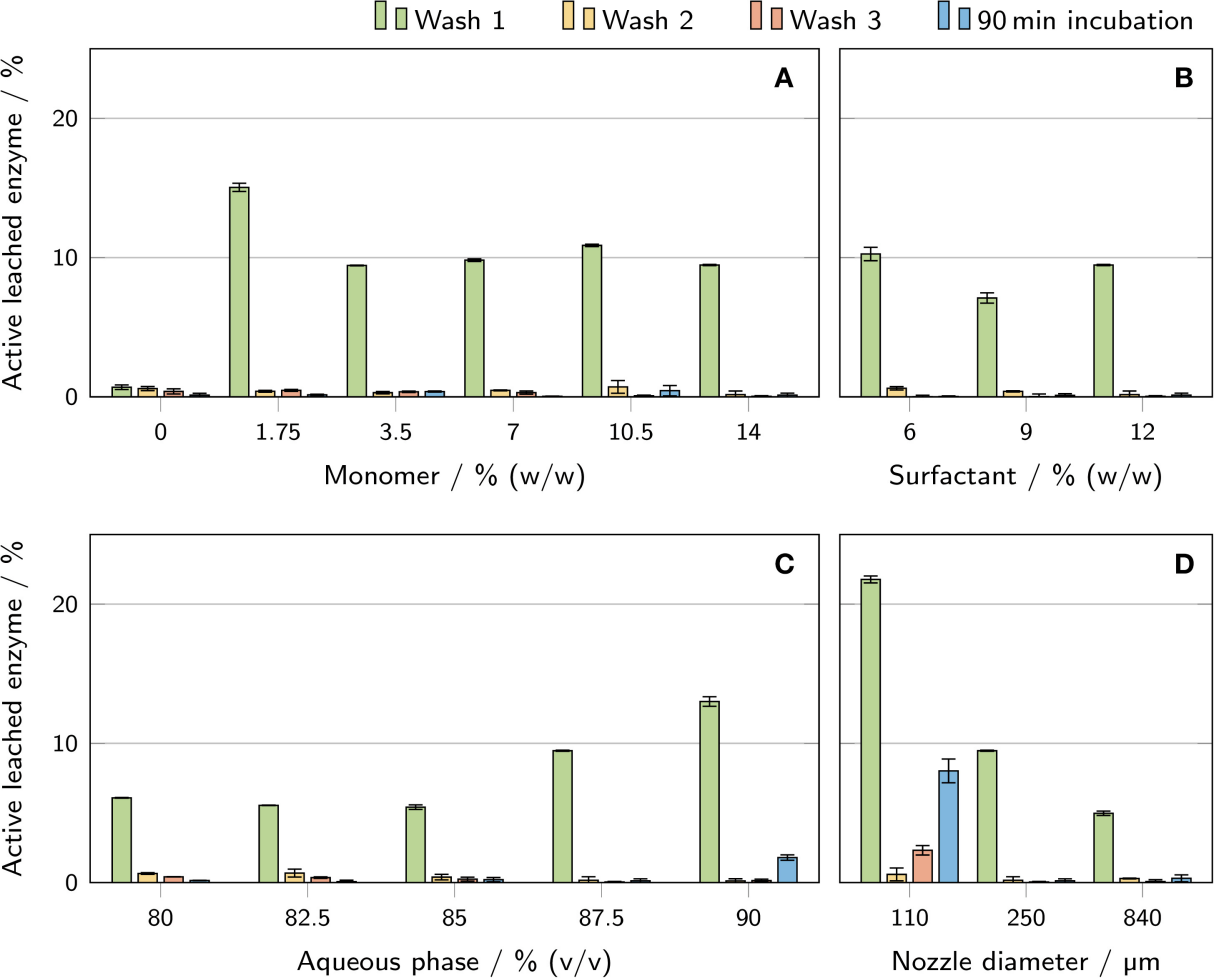
Determination of the amount of active enzyme leached from the printed polyHIPE cylinders during the wash procedure and a 90 min incubation in a microtiter plate. The results are presented as mean values ± standard deviation (*n* = 3). The influence of **(A)** monomer mass fraction in the aqueous phase, **(B)** surfactant mass fraction in the organic phase, **(C)** aqueous phase volume fraction, and **(D)** nozzle diameter is shown.

In order to quantify the amount of active enzyme leached from the polyHIPE cylinders during the three wash steps and a 90 min incubation period, activity assays were conducted with the supernatants of the wash and incubation steps (Figure 8). The most remarkable result was found for wash step 1 of the polyHIPE sample with a monomer concentration of 0% (w/w) in the aqueous phase. Here, the determined amount of leached active enzyme is only a sixth of the second lowest value determined for step 1. Furthermore, this was the only sample exhibiting a similar amount of leached active enzyme for all three wash steps. For all other conditions, the amount of active enzyme leached during the first step was at least 4-fold higher than for the two subsequent steps. A clear trend could be observed for the volume fraction of aqueous phase where a higher volume fraction correlated with a higher amount of active leached enzyme in the supernatant of wash step 1. The relative loss of enzyme during the first wash step was also inversely correlated with the nozzle diameter. However, with a maximum deviation of 6% between the samples, the absolute amount of leached enzyme was nearly identical for all three nozzle diameters. Regarding the supernatants of the 90 min incubation samples, only two showed more than 0.5% leached enzyme: (1.8 ± 0.2)% for samples with 90% aqueous phase volume fraction and (8.0 ± 0.9)% for samples printed with 110 μm nozzles.

### 3.6. Activity Assays

Activity assays with printed hydrogel-filled polyHIPE cylinders containing β-galactosidase in the aqueous phase were performed to investigate the influence of various parameters on the resulting enzymatic activity. All values discussed here are specific activity values calculated per mg of β-galactosidase crude cell extract from *Aspergillus oryzae*. Figure 3 shows the typical evolution of absorbance (Figure 3A) and product concentration (Figure 3B) over time for different substrate concentrations. Unlike in assays with freely dissolved enzyme, the maximum turnover rates did not occur immediately after substrate addition but after a delay of several minutes with steadily increasing turnover rates. The maximum turnover rate of each sample was determined and the specific activity calculated to ensure comparability between experiments with different amounts of enzyme. Plotting the specific activities over substrate concentration resulted in curves resembling Michaelis-Menten kinetics which were fitted accordingly (Figure 3C). Combining different kinetics curves with a 3D surface plot allows depicting variations in activity over substrate concentration and a second parameter (Figure 3D).

**Figure 9:**
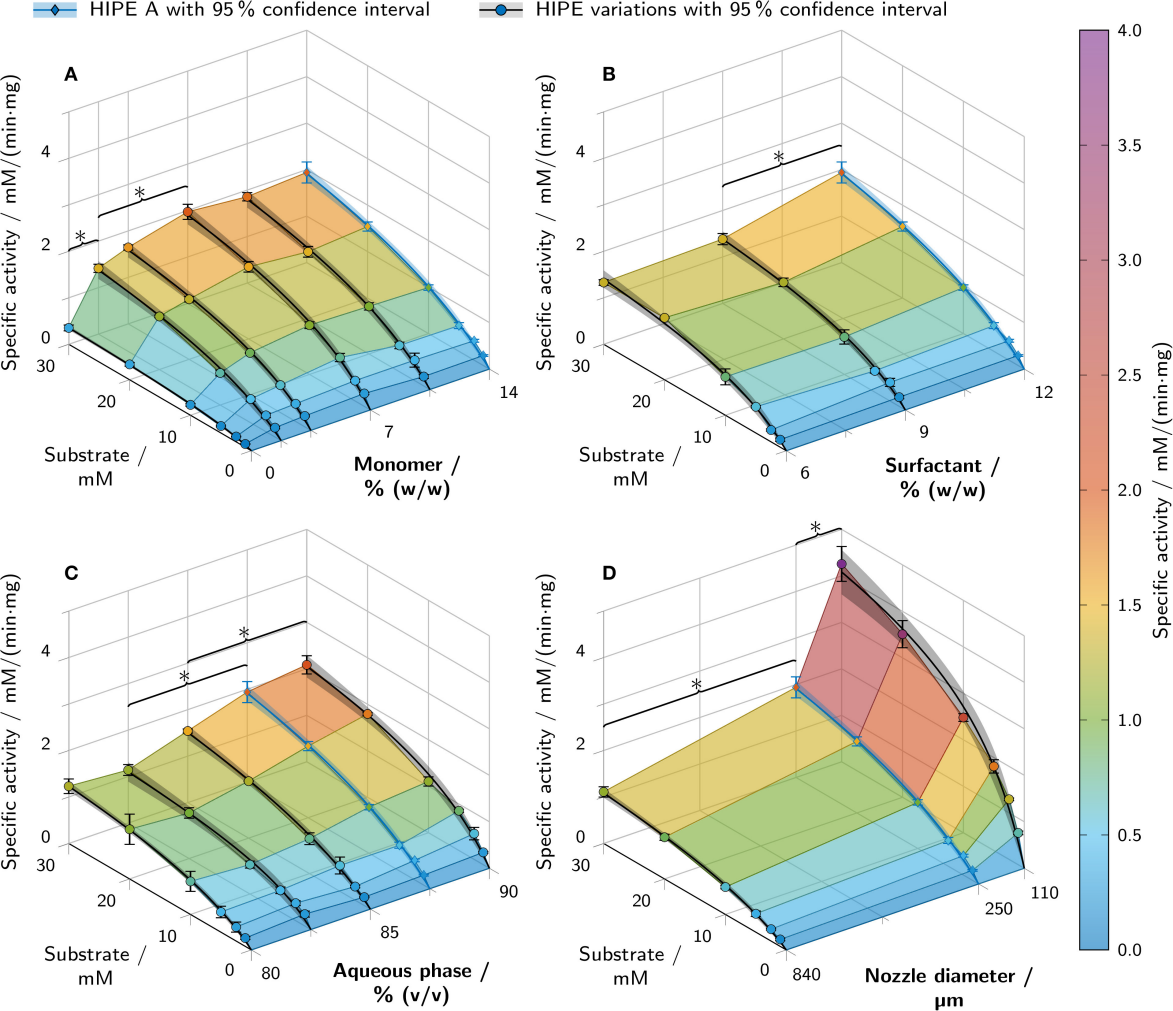
Results of batch activity screenings of printed polyHIPE cylinders containing β-galactosidase. The apparent specific activity is shown over the substrate concentration and a second varied parameter. All variations are derived from HIPE A printed with a 250 μm nozzle (depicted in blue in each graph). Varied parameters were **(A)** the monomer mass fraction in the aqueous phase, **(B)** the surfactant mass fraction in the organic phase, **(C)** the aqueous phase volume fraction and **(D)** the nozzle diameter. Data points are represented as mean ± standard deviation (*n* = 3). The black and blue lines represent Michaelis-Menten fits with 95% confidence intervals in semi-transparent shading. For clarity, only significant differences to the nearest significantly different data points at 30 mM substrate are highlighted by asterisks (*p* < 0.05).

The resulting kinetics of all performed activity assays are presented in Figure 9. Of the three HIPE composition parameters being varied, the monomer mass fraction in the aqueous phase had the highest impact on specific activity. An increase in monomer mass fraction from 0% to 7% correlated with a more than 5-fold increase in specific activity at 30 mM substrate concentration (Figure 9A). Increasing the amount of monomer further to 14% (w/w) had no significant additional effect. PolyHIPEs with 12% (w/w) surfactant in the organic phase showed a 1.5 times higher specific activity at 30 mM substrate than polyHIPEs with 9% or 6% (w/w) surfactant (Figure 9B). The aqueous phase volume fraction had an influence on the specific activity at 30 mM substrate in the range between 82.5% and 87.5% (v/v) with a 1.7-fold increase (Figure 9C). Below 82.5% and above 87.5%, no further significant changes could be observed. Besides the variations of HIPE composition, HIPE A was printed using nozzles with different diameters in order to evaluate the influence of cylinder thickness on the resulting specific activity (Figure 9D). The lowest activity at 30 mM substrate was determined for a nozzle diameter of 840 μm with a value of (1.1 ± 0.1) mM/(min· mg). In comparison, cylinders printed with a 250 μm nozzle showed a nearly 2-fold increase in specific activity and a nearly 4-fold increase was determined for the 110 μm nozzle. This was also the highest specific activity of all analyzed samples, more than two times higher than any other condition.

### 3.7. Error Analysis of PolyHIPE Activity Assays

An error estimation with a worst-case scenario (error *E*_*max*_) and a best-case scenario (error *E*_*min*_) was performed in order to estimate the proportion of activity observed in the polyHIPE activity assays that was caused by leached rather than immobilized enzyme, as described in section 2.10. The highest *E*_*max*_ of 12.79% was found for polyHIPEs printed with 110 μm nozzles, *E*_*min*_ was 1.28%. PolyHIPEs with 90% (v/v) aqueous phase volume fraction showed the second highest *E*_*max*_ with 5.85%. All other samples showed values between 0.14% and 2.30% for *E*_*max*_ and between 0.03% and 1.74% for *E*_*min*_, as listed in Table S3.

## 4. Discussion

This study aims at formulating aqueous solutions of AA and PEG-DA 700 as the internal phase of HIPEs in order to enhance printability and allow the 3D printing of composite materials consisting of porous polymeric scaffolds filled with enzyme-laden hydrogels. Different HIPE compositions were systematically characterized to assess rheological and morphological properties, equilibration time as a measure of diffusion behavior, leaching and enzymatic activity.

### 4.1. Rheology and Printability

Yield stress is an essential predictor of printability, especially for structures involving overhangs, as it substantially determines the bridging and shape retention capabilities of the material after extrusion (Sears et al., 2019). While the aqueous phase volume fraction and the surfactant concentration in the organic phase were found to strongly influence the yield stress, the monomer concentration in the aqueous phase had a minor effect (Figure 4). This indicates that little or no adverse effects on the printability of HIPEs are to be expected from the addition of AA and PEG-DA 700 to the aqueous phase. However, high surfactant concentrations and high aqueous phase volume fractions are desirable to enhance printing quality and allow the fabrication of more complex geometries. Shear stress-controlled oscillatory measurements indicated a gel-like character for all samples and could confirm the trends observed for yield stress.

The void size of the prepared polyHIPEs was found to be inversely correlated to the surfactant concentration in the organic phase (Figure 6). These results are in accordance with results described by Princen who showed that the yield stress of HIPEs is a function of aqueous phase volume fraction, droplet size and interfacial tension (Princen, 1983, 1985). HIPE A, one of the HIPEs with a high yield stress, exhibited excellent printability, as demonstrated by the printed grid and gyroid structures shown in Figure 5. High-quality prints are essential to reduce mass transfer limitations in biocatalytic reactors, as they enable the fabrication of flow-optimized geometries with thin walls (Schmieg et al., 2019).

### 4.2. PolyHIPE Morphology

It was expected that the addition of AA and PEG-DA 700 as monomers and LAP as a photoinitiator to the aqueous phase of the HIPEs would result in polyHIPE scaffolds filled with hydrogel. This was intended in order to retain the enzyme within the material and prevent it from leaching through the interconnecting pores of the polyHIPE while still allowing the diffusion of the relatively small substrate and product molecules. ESEM micrographs (Figures 6G–I) revealed that most voids of samples with 14% (w/w) monomer in the aqueous phase were indeed filled, while the control samples with 0% (w/w) monomer showed empty voids. This implies a successful formation of hydrogel inside the voids. Previous studies about hydrogel-filled polyHIPEs have addressed aspects like monomer content in the aqueous phase and locus of initiation (Gitli and Silverstein, 2008), pre-polymerization of the organic phase (Gitli and Silverstein, 2011), degree of cross-linking (Kovačič et al., 2011) or shape-memory properties (Warwar Damouny and Silverstein, 2016). These studies mostly used scanning electron microscopy (SEM) micrographs of dried samples for morphology analysis and typically found bi-continuous systems with an interconnected hydrogel that collapsed onto the polyHIPE walls covering the interconnecting pores of the polyHIPE scaffold. Also, depending on locus of initiation and monomer content in the aqueous phase, hydrogels covering only the polyHIPE walls while leaving the interconnecting pores open were described (Gitli and Silverstein, 2008). However, all of the cited studies were conducted with styrene-based organic phases, thermal initiators and different surfactants than used here, so comparability is limited. The SEM and ESEM micrographs of the samples prepared in this study show mostly separate hydrogel spheres, not connected with the hydrogel in neighboring voids or the surrounding polyHIPE scaffold. This may imply the presence of a liquid interface layer between hydrogel and surrounding polyHIPE which could be beneficial for mass transport, as it offers an alternative route through the composite material avoiding passage through the hydrogel phase. The high number of empty voids found in SEM as opposed to ESEM micrographs can probably be attributed to the sample preparation method which in the case of SEM involves a freeze-drying step causing the hydrogel to shrink and dry out which may promote the detachment of the hydrogel from the scaffold. ESEM samples were prepared in a hydrated state which may have caused an increased adherence of the hydrogel to the polyHIPE scaffold and hence a reduced loss. The observed shrinkage of hydrogel spheres in ESEM samples can be attributed to a drying effect due to the exposure to air directly after sample preparation.

The decreased stability of the polyHIPE scaffold with 12% (w/w) surfactant in the organic phase and 14% (w/w) monomer in the aqueous phase may be caused by the incorporation of water-soluble monomers into the external scaffold which has been reported to have a destabilizing effect leading to the collapse of the scaffold upon drying (Gitli and Silverstein, 2008, 2011). The intact polyHIPE morphology of printed samples (see Figure S2) shows the suitability of HIPEs for the application in extrusion-based 3D printing.

### 4.3. Mass Transfer Limitations

Immobilizing enzymes in hydrogel-filled polyHIPEs may result in two main disadvantages: reduced mass transfer caused by the hydrogel and the polyHIPE scaffold and stress-induced enzyme inactivation due to harsh conditions during HIPE preparation. As a measure of mass transfer limitations, ONP solutions were added to the printed polyHIPE cylinders and the equilibration time was determined (Figure 7). A shorter equilibration time can be attributed to a higher mass transfer through the material or a shorter path length due to a thinner cylinder. Both enable the embedded enzyme to work more efficiently due to an improved substrate supply.

No clear influence on equilibration time was found for the monomer mass fraction in the aqueous phase. However, both a higher surfactant mass fraction in the organic phase and a higher aqueous phase volume fraction correlated with a shorter equilibration time. Assuming that all cylinders had approximately identical dimensions, it can be concluded that polyHIPEs with a higher amount of surfactant or internal phase allow a higher mass transfer through the material. This may be caused by an increased degree of openness of the polyHIPE scaffold. The degree of openness is the ratio of open surface to total surface of a scaffold cavity and typically increases with both surfactant concentration and aqueous phase volume fraction (Pulko and Krajnc, 2012). Assuming that substrate and product molecules are able to diffuse through the internal hydrogel phase but not through the polyHIPE scaffold itself, a high degree of openness would allow for a less tortuous and hence shorter path through the material. This may explain the decreased equilibration time for samples with high amounts of surfactant and aqueous phase observed in this study.

As discussed in section 4.2, a second pathway through a liquid-filled interface between hydrogel and polyHIPE scaffold may be available for a more efficient mass transport. Due to the absence of hydrogel, this pathway would offer a higher rate of diffusion for both substrate and product but a higher tortuosity due to the necessity of bypassing the hydrogel. Mass transport via this pathway would benefit from a smaller void size and hence decreased tortuosity. As has been shown here (Figure 6) and in other studies (Williams et al., 1990; Zhang et al., 2019), smaller void sizes can be achieved by higher amounts of surfactant.

The lowest equilibration time was determined for cylinders printed with a 110 μm instead of a 250 μm nozzle. Here, the low equilibration time can be attributed to a lower path length through the cylinder which demonstrates the importance of printing fine structures to reduce mass transfer limitations.

### 4.4. Apparent Enzymatic Activity, Leaching, and Enzyme Inactivation

The main focus of this work was to establish printable HIPE formulations as bioinks for enzymes and to evaluate the effect of different HIPE compositions and nozzle diameters on the apparent enzymatic activity of the printed and polymerized material. Besides mass transfer limitations, stress-induced enzyme inactivation and leaching during the wash steps may cause a reduced apparent activity. Potential sources of stress-induced enzyme inactivation in the applied process are the contact with highly hydrophobic organic compounds in the organic phase (Iyer and Ananthanarayan, 2008) or monomer species in the aqueous phase, high shear forces during the emulsification process (Bekard et al., 2011) or UV irradiation (Luse and McLaren, 1963; Vladimirov et al., 1970), free radicals (Dumitru and Nechifor, 1994) and increased temperature (Ustok et al., 2010) during the exothermic photo-crosslinking reaction. The measured activities alone cannot give any indication about the predominant aspect leading to reduced apparent activity. However, in combination with the determined equilibration time (Figure 7) as a measure of mass transfer limitations and the amount of active enzyme leached during the wash procedure (Figure 8), certain conclusions can be drawn about the causes for activity differences of different samples.

For both surfactant mass fraction in the organic phase and aqueous phase volume fraction, a decline in equilibration time (Figures 7B,C) correlated with an increase in apparent activity (Figures 9B,C). This indicates that the change in apparent activity for these parameters can at least partly be explained by differences in mass transfer limitations of the polyHIPE compositions. In contrast, the monomer mass fraction in the aqueous phase had no significant effect on the equilibration time while still influencing the apparent activity between 0% and 14% (w/w) monomer by a factor of 5. In this case, the reduced activity at 0% monomer cannot be attributed to mass transfer limitations. A reasonable assumption would be that the enzyme is lost during the wash steps due to the lack of a retaining hydrogel, but analysis of the wash solutions showed a decreased rather than increased amount of leached active enzyme in these samples (see Figure 8). This eliminates the leaching of active enzyme as a cause for the decrease in apparent activity at lower amounts of monomer in the aqueous phase and leaves enzyme inactivation as the most probable explanation. Depending on concentration and chain length, PEG is known to stabilize proteins in aqueous solutions (Wang, 1999), so the presence of PEG-DA 700 might preserve the enzymatic activity of the β-galactosidase during the emulsification and polymerization process.

A very simple and efficient way to reduce mass transfer limitations is to decrease diffusion path lengths by increasing the surface-area-to-volume ratio of the printed material (Schmieg et al., 2018), as demonstrated here by the equilibration time analysis of cylinders printed with different nozzles (Figure 7D). In an enzymatic reactor, this can be realized in the form of finer grids. Hence, the influence of printing with different nozzle diameters was investigated to determine the achievable increase in apparent specific activity. It should be noted that the nozzle diameter does not correspond to the thickness of the printed strand but is also dependent on printing parameters like extrusion rate and layer height. Using 110 μm instead of 840 μm nozzles caused a 5-fold increase in apparent activity at 30 mM ONPG for an identical polyHIPE composition (see Figure 9). This demonstrates that decreasing the diffusion path length and increasing the surface-area-to-volume ratio is an essential prerequisite for efficient biocatalytical processes.

Regarding leaching behavior, all polyHIPEs containing hydrogel lost a significant amount of enzyme, especially during the first wash step (see Figure 8). To avoid this, enzymes could be covalently incorporated into the hydrogel with appropriate linker molecules. During the 90 min incubation period following the wash steps, most samples lost less than 0.5% of enzyme. However, the cylinders printed with a 110 μm nozzle lost an amount of enzyme several times higher than all other samples. Due to their thin strands, these cylinders were more flexible than others and underwent higher mechanical deformations during the wash process and transfer to microtiter plates. This stress may have caused defects in the polyHIPE scaffold leading to increased leaching of enzyme or enzyme-containing hydrogel particles. The influence of leached enzyme on the results of the polyHIPE activity assays is discussed in the following section.

### 4.5. Error Analysis of PolyHIPE Activity Assays

Results of activity assays with enzyme immobilized in polyHIPEs could be influenced by leached enzyme. An error estimation with a worst-case scenario (error *E*_*max*_) and a best-case scenario (error *E*_*min*_) was performed in order to estimate the proportion of the observed activity that was caused by leached rather than immobilized enzyme. The calculation is based on the volumetric activity of supernatant samples incubated for 90 min with a polyHIPE cylinder. The worst-case scenario assumes the leached enzyme to be present from the beginning of the activity assay reaction while the best-case scenario assumes a linear release profile over 90 min, as described in section 2.10. Hence, *E*_*max*_ only depends on the volumetric activity of the supernatant after a 90 min incubation and the maximum activity occurring during the polyHIPE activity assay, while *E*_*min*_ is also proportional to the time delay until the maximum activity occurs.

Due to the high amount of leached enzyme during the 90 min incubation, a high *E*_*max*_ of 12.79% was found for polyHIPEs printed with 110 μm nozzles. The high discrepancy between *E*_*max*_ and *E*_*min*_ (1.28%) can be attributed to the short diffusion path lengths in these samples allowing the maximum activity to occur after a short time delay of 9 min after substrate addition. For all other samples, *E*_*max*_ was below 6% and *E*_*min*_ below 2%. Further experiments would be necessary to determine a realistic release profile instead of a best- and worst-case scenario. However, even assuming the worst-case scenario, errors can be neglected for most samples and all trends observed in the polyHIPE activity assays (Figure 9) can be confirmed. The observed differences in activity can indeed be attributed to the polyHIPE material properties, not different amounts of leached enzyme in the supernatant.

### 4.6. Conclusion

This study demonstrates the applicability of HIPEs as enzyme-containing bioinks for the extrusion-based 3D printing of enzymatically active composite materials to be employed in biocatalytic reactors. Most prepared HIPEs exhibited excellent printability allowing the fabrication of complex 3D structures without the need for sacrificial support material. The inks could be polymerized employing a cure-on-dispense setup resulting in polyHIPE scaffolds filled with hydrogel, as confirmed by (E)SEM micrographs. Automated activity assays showed that β-galactosidase could be preserved within the polyHIPEs in an active state and that it could be supplied with the substrate ONPG. The presented activity assay method allows the time-efficient screening of a large variety of materials, printing parameters and reaction conditions enabling a systematic optimization with regard to apparent activity. Mass transfer limitations and enzyme inactivation were identified as the most important factors limiting the apparent activity. While not significantly affecting the mass transfer of the material, the presence of monomer in the aqueous phase was found to be essential for the preservation of enzymatic activity: PolyHIPEs with 14% (w/w) monomer showed a more than fivefold higher apparent activity than polyHIPEs with 0% (w/w) monomer in the aqueous phase. Printing thin structures was shown to reduce diffusion path lengths and hence mass transfer limitations, causing a nearly fourfold increase in specific apparent activity for prints from 110 μm nozzles, as compared to 840 μm nozzles. The surfactant mass fraction in the organic phase and the aqueous phase volume fraction were found to have a less pronounced but still significant effect on mass transfer and hence apparent activity. Due to their excellent printability, the presented bioinks are suitable for the additive manufacturing of more sophisticated and finer resolved biocatalytic reactor designs with reduced mass transfer limitations.

In order to reduce mass transfer limitations even further, oil-in-water instead of water-in-oil HIPEs could be produced with an external enzyme-laden hydrogel phase and an internal phase consisting of inert oil droplets. However, these materials are likely to exhibit relatively low mechanical stability and may require time-consuming wash steps in order to remove the internal phase. An alternative field of use for the materials presented in this study could be sustained release applications with tunable release kinetics by altering both HIPE composition and the geometry of the print.

## Data Availability Statement

The raw data supporting the conclusions of this article will be made available by the authors, without undue reservation.

## Author Contributions

LW, MW, and JH conceptualized the research. LW developed the employed lab ware, established automated data evaluation tools, conducted all shown experiments and wrote the manuscript. CR provided expertise on automated activity assays and bioprinting, MW expertise on HIPEs. JG established workflows for HIPE preparation and printing. JG and LW established the activity assay procedure. CR, MW, and JH proofread the manuscript. All authors contributed to the article and approved the submitted version.

## Conflict of Interest

The authors declare that the research was conducted in the absence of any commercial or financial relationships that could be construed as a potential conflict of interest.
