# Supplementary material for: 3D-Printable and Enzymatically Active Composite Materials Based on Hydrogel-Filled High Internal Phase Emulsions
Source: Front Bioeng Biotechnol. 2020 Jul 21;8:713. doi: 10.3389/fbioe.2020.00713 (PMC7396703; doi:10.3389/fbioe.2020.00713)
Supplement: Supplementary file 1 [file Data_Sheet_1.pdf]

Supplementary Material

1 MATERIALS AND METHODS

1.1 HIPE preparation

Table S1. Compositions of organic phase used in this study. Most HIPEs were prepared with a concentration of 12 % (w/w) surfactant (Pluronic® L-121).

EHA (% (w/w))	IBOA (% (w/w))	TMPTA (% (w/w))	Pluronic® L-121 (% (w/w))	Darocur® TPO (% (w/w))
26.9	48.9	11.7	12.0	0.5
27.8	50.6	12.1	9.0	0.5
28.7	52.2	12.5	6.0	0.5

Table S2. Parameters for the preparation of HIPEs with different aqueous phase volume fractions. The amounts of organic and aqueous phase were adapted in order to always produce 20 mL of HIPE in total. The addition rate of aqueous phase was adapted relative to the amount of organic phase and the time points of stirring rate changes were adapted in relation to the duration of the addition process.

				Time points of stirring rate changes	
Aqueous phase volume fraction (% (v/v))	Amount of organic phase (mL)	Amount of aqueous phase (mL)	Addition rate (mL/min)	To 800 rpm (min)	To 1000 rpm (min)
90	2	18	1.00	2.6	9.0
87.5	2.5	17.5	1.25	2.0	7.0
85	3	17	1.50	1.6	5.7
82.5	3.5	16.5	1.75	1.3	4.7
80	4	16	2.00	1.1	4.0

1.2 Determination of equilibration time

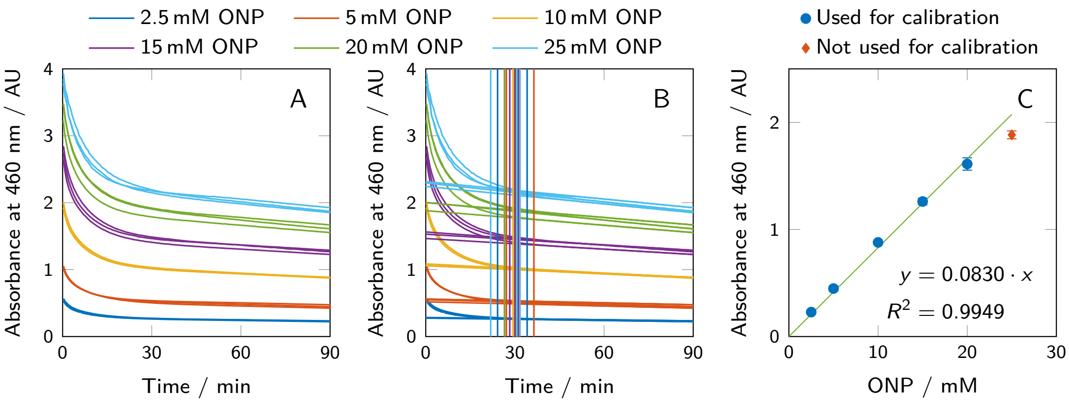

Figure S1: (A) Recorded raw data for ONP calibration curves and the determination of equilibration time of HIPE A. Buffered solutions of different concentrations of ONP were added to printed polyHIPE cylinders and the absorbance at 460 nm was measured for 90 min. (B) To determine the equilibration time, the last 30 min of each curve were fitted with a linear equation and the equilibration time was determined by calculating the time point at which the slope averaged over 5 min was within a 1.2-fold range of the end slope (resulting time points indicated by vertical lines). The resulting data were used to generate the equilibration time box plots. (C) ONP calibration curves were determined from the end points of the raw data in a range from 2.5 mM to 20 mM.

2 RESULTS

2.1 PolyHIPE morphology

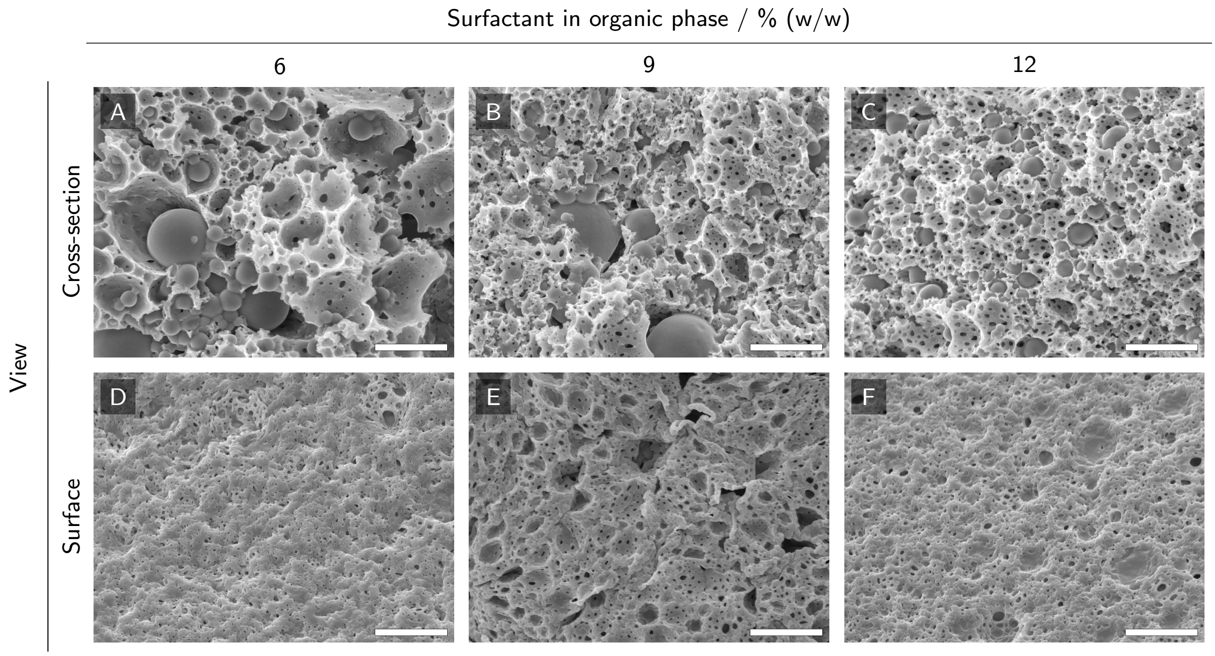

Figure S2: (E)SEM micrographs of polyHIPEs printed with a 250 µm conical nozzle. Samples with different surfactant mass fractions in the organic phase are shown. (A-C) View of the cross-section. (D-F) View of the surface. The scale bars represent 10 µm.

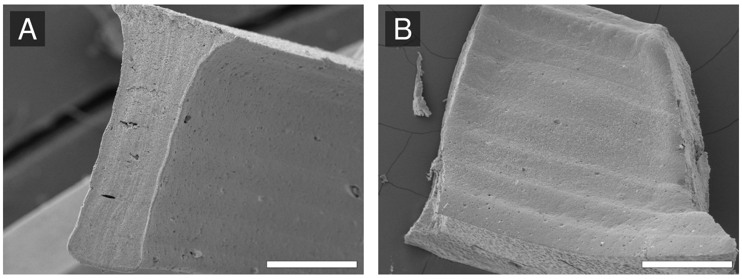

Figure S3: (E)SEM micrographs of a polyHIPE cylinder printed with a 250 µm nozzle. (A) View of the cross-section. (B) View of the inner surface of the cylinder. The scale bars represent 500 µm.

2.2 Activity assay error estimation

Table S3. Error estimations analyzing the influence of leached enzyme on the performed activity assays with printed polyHIPE cylinders. Errors for best case and worst case scenarios are calculated based on the apparent volumetric activity $v_{apparent}$ during the polyHIPE activity assay and the volumetric activity of the supernatant after an incubation period of 90 min ($v_{leached,\,90\,\mathrm{min}}$). The values for $v_{leached,\,90\,\mathrm{min}}$ are already corrected for the dilution occuring during the supernatant activity assay.

			Volumetric activity		Error	
		Delay until max. activity t_{delay} (min)	Total (observed) $v_{apparent}$ (mM/min)	Supernatant after 90 min $v_{leached,\,90\,\mathrm{min}}$ (mM/min)	Worst case E_{max} (%)	Best case E_{min} (%)
Monomer (% (w/w))	14	22.2	0.268	1.2×10^{-3}	0.43	0.11
	10.5	20.1	0.256	4.2×10^{-3}	1.62	0.36
	7	19.5	0.272	3.9×10^{-4}	0.14	0.03
	3.5	30.8	0.227	3.6×10^{-3}	1.57	0.54
	1.75	29	0.195	1.4×10^{-3}	0.71	0.23
	0	68.1	0.049	1.1×10^{-3}	2.30	1.74
Surfactant (% (w/w))	12	22.2	0.268	1.2×10^{-3}	0.43	0.11
	9	31.2	0.191	1.0×10^{-3}	0.54	0.19
	6	31.9	0.184	2.5×10^{-4}	0.14	0.05
Aqueous phase (% (v/v))	90	21.3	0.296	1.7×10^{-2}	5.85	1.38
	87.5	22.2	0.268	1.2×10^{-3}	0.43	0.11
	85	38	0.207	1.9×10^{-3}	0.93	0.39
	82.5	33.7	0.149	5.8×10^{-4}	0.39	0.14
	80	22.6	0.157	1.3×10^{-3}	0.82	0.21
Nozzle diameter (µm)	840	23.2	0.249	4.7×10^{-3}	1.89	0.49
	250	22.2	0.268	1.2×10^{-3}	0.43	0.11
	110	9	0.244	3.1×10^{-2}	12.79	1.28
